# Analysis of miRNAs Profiles in Serum of Patients With Steatosis and Steatohepatitis

**DOI:** 10.3389/fcell.2021.736677

**Published:** 2021-09-09

**Authors:** Maria Vulf, Daria Shunkina, Aleksandra Komar, Maria Bograya, Pavel Zatolokin, Elena Kirienkova, Natalia Gazatova, Ivan Kozlov, Larisa Litvinova

**Affiliations:** ^1^Center for Immunology and Cellular Biotechnology, Immanuel Kant Baltic Federal University, Kaliningrad, Russia; ^2^Department of Organization and Management in the Sphere of Circulation of Medicines, Institute of Postgraduate Education, I.M. Sechenov Federal State Autonomous Educational University of Higher Education—First Moscow State Medical University of the Ministry of Health of the Russian Federation (Sechenov University), Moscow, Russia

**Keywords:** miRNA, mRNA, NAFLD, steatosis, NASH, GSEA, KEGG, REACTOME

## Abstract

Non-alcoholic fatty liver disease (NAFLD) is emerging as one of the most common chronic liver diseases worldwide, affecting 25% of the world population. In recent years, there has been increasing evidence for the involvement of microRNAs in the epigenetic regulation of genes taking part in the development of steatosis and steatohepatitis—two main stages of NAFLD pathogenesis. In the present study, miRNA profiles were studied in groups of patients with steatosis and steatohepatitis to compare the characteristics of RNA-dependent epigenetic regulation of the stages of NAFLD development. According to the results of miRNA screening, 23 miRNAs were differentially expressed serum in a group of patients with steatohepatitis and 2 in a group of patients with steatosis. MiR-195-5p and miR-16-5p are common differentially expressed miRNAs for both steatosis and steatohepatitis. We analyzed the obtained results: the search for target genes for the differentially expressed miRNAs in our study and the subsequent gene set enrichment analysis performed on KEGG and REACTOME databases revealed which metabolic pathways undergo changes in RNA-dependent epigenetic regulation in steatosis and steatohepatitis. New findings within the framework of this study are the dysregulation of neurohumoral pathways in the pathogenesis of NAFLD as an object of changes in RNA-dependent epigenetic regulation. The miRNAs differentially expressed in our study were found to target 7% of genes in the classic pathogenesis of NAFLD in the group of patients with steatosis and 50% in the group of patients with steatohepatitis. The effects of these microRNAs on genes for the pathogenesis of NAFLD were analyzed in detail. MiR-374a-5p, miR-1-3p and miR-23a-3p do not target genes directly involved in the pathogenesis of NAFLD. The differentially expressed miRNAs found in this study target genes largely responsible for mitochondrial function. The role of miR-423-5p, miR-143-5p and miR-200c-3 in regulating apoptotic processes in the liver and hepatocarcinogenesis is of interest for future experimental studies. These miR-374a, miR-143, miR-1, miR-23a, and miR-423 have potential for steatohepatitis diagnosis and are poorly studied in the context of NAFLD. Thus, this work opens up prospects for further studies of microRNAs as diagnostic and therapeutic biomarkers for NAFLD.

## Introduction

Currently, non-alcoholic fatty liver disease (NAFLD) is emerging as one of the most common chronic liver diseases worldwide, affecting 25% of the world population, with the highest prevalence in the Middle East and South America (Younossi et al., 2019). The prevalence of NAFLD in the population ranges from 6.3 to 33.0%, with a higher prevalence in obese individuals (up to 62–93%). NAFLD currently occupies a leading place among internal diseases of Russian Federation (Lazebnik et al., 2021). In Russia, the prevalence of NAFLD among the adult population is 37% (Maev et al., 2019).

Non-alcoholic fatty liver disease is a disease spectrum that initially develops from hepatic steatosis and progresses to non-alcoholic steatohepatitis (NASH), cirrhosis, and eventually hepatocellular carcinoma (HCC). NAFLD is characterized by the accumulation of fatty deposits in the hepatocyte parenchyma caused by increased triglyceride (TG) formation against a background of elevated free fatty acids (FFA) and impaired regulation of *de novo* lipogenesis. Excessive FFA accumulation provokes oxidative stress and increases the content of reactive oxygen and nitrogen species. Formation of lobular inflammation and hepatocellular ballooning are signs of the NASH stage. Against the background of the inflammatory response, hepatic stellate cells (HSCs) are activated and transformed into myofibroblasts, leading to liver fibrosis (Gazatova et al., 2019). Numerous factors are involved in the pathogenesis of NAFLD. However, the complete picture of NAFLD development and the transition mechanisms from steatosis to NASH are not yet fully understood.

Non-alcoholic fatty liver disease is nowadays diagnosed by invasive methods (e.g., histological), the diagnostic accuracy of minimally invasive methods is not optimal (Lackner, 2021). In the later stages of NAFLD, it is only possible to slow but not prevent the transformation to HCC, which requires liver transplantation. Researchers predict that HCC will be the leading cause of liver transplantation worldwide by 2025 (Llovet et al., 2021). In fact, from 2002 to 2016, the proportion of liver transplants caused by NAFLD and its complications increased from 1.2 to 8.4% of all liver transplants (Haldar et al., 2019). Because NAFLD ultimately leads to disability and imposes a major socioeconomic burden, timely diagnosis and effective treatment of NAFLD is particularly important.

One of the significant diagnostic as well as therapeutic targets for NAFLD are small non-coding RNAs (ncRNAs). Thousands of them have been found in the human genome, including microRNAs (miRNAs). MiRNAs are small non-coding 21–23 nucleotide long molecules involved in post-transcriptional regulation of gene expression (Skuratovskaia et al., 2019). These RNAs consist of an inactive sense strand complementary to the target mRNA sequence and an active antisense strand (Rana, 2007).

The use of ncRNAs is a promising epigenetic approach to target the major factors influencing the development of NAFLD: impaired lipid and carbohydrate metabolism, the development of oxidative stress, and chronic subclinical inflammation. Despite the wide range of drugs on the market, the risk of developing complications of this pathology remains at a high level. Therefore, it is important to study the molecular basis of this disease.

The present study aims to compare miRNA profiles in patients with steatosis and steatohepatitis using bioinformatics and experimental methods and assess their effect on NAFLD progression.

## Materials and Methods

**FIGURE 1:**
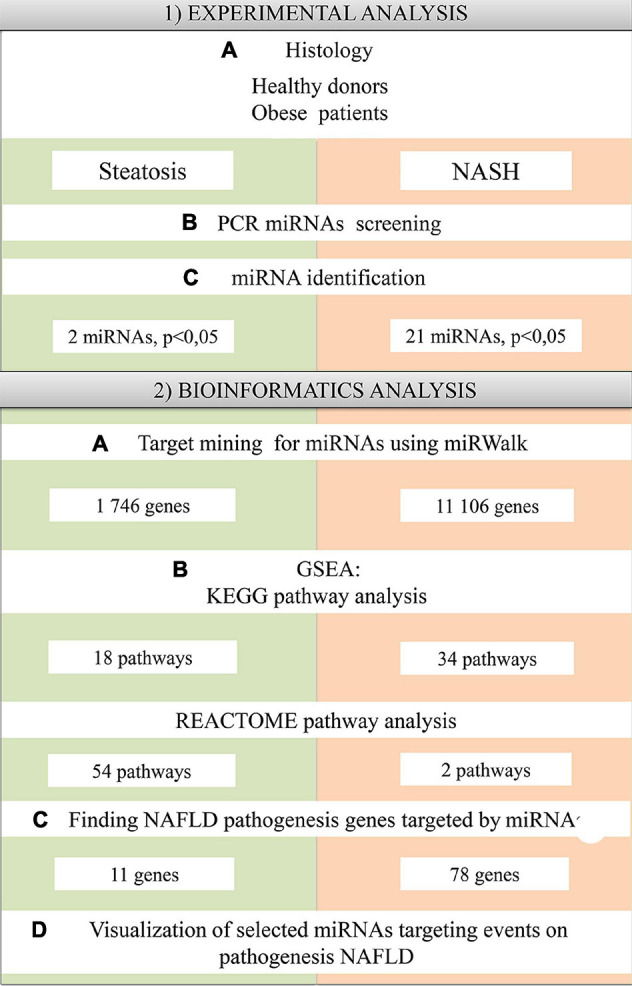
Schematic representation of the study design.

Sixteen patients aged 18 to 65 years (37.7 ± 9.7 years; seven men, nine women) were included in the study. eight patients with morbid obesity were diagnosed with NAFLD with steatosis/NASH (42.6 ± 9.9 years; five men, seven women). These samples were compared with four healthy donors (32.5 ± 9.5 years; two men, two women) (Table 1). Exclusion criteria were: age up to 18 years and over 65 years; the presence of infectious liver disease; acute and severe chronic somatic and infectious concomitant diseases; long-term use of lipid-lowering drugs; and patients who refused medical and laboratory controls during the study. Patients were monitored in a hospital (Regional Clinical Hospital of Kaliningrad Region). According to the DEBQ Dutch Eating Behavior Questionnaire, patients provided information about their diet (van Strien et al., 1986). Patients underwent standard dietary adjustments prior to surgery as all obese patients underwent bariatric surgery while the healthy donors underwent elective surgery with general anesthesia. According to the Moorehead-Ardelt Quality of Life Questionnaire II, the obese patients had low activity levels (Myers et al., 2006). A blood sample is taken in the morning on an empty stomach before surgery. A block diagram of the study design is shown in Figure 1.

**TABLE 1:** Characteristics of patients in the study.

	Healthy donors, *n* = 4	Group of patients with steatosis, *n* = 6	Group of patients with NASH, *n* = 6
	
	1	2	3
Sex (female/male)	2/2	3/3	4/2
Age	32.50 ± 9.50	41.75 ± 9.91	43.50 ± 11.50
BMI, kg/m^2^	21.88 ± 0.66	51.05 ± 10.04 p 1-2**	42.95 ± 4.67 p 1-3***

**p < 0.001, ***p < 0.0001; significance was determined using the *t*-test (mean ± SD).

All study participants gave informed consent to participate in the study. The study was conducted in accordance with the World Medical Association Declaration of Helsinki (2000) and the Protocol to the Convention on Human Rights and Biomedicine (1999). Local Ethics Committee of the Immanuel Kant Baltic Federal University, Protocol No. 4 of November 29, 2018.

### Histological Analysis

Histological analysis of liver biopsies was performed to confirm steatosis and NASH. Liver biopsies obtained intraoperatively by incisional biopsy from the left lobe of the liver were fixed in neutral buffered formalin. Paraffin sections of the liver with a thickness of 4–5 μm, stained with hematoxylin and eosin, were studied by traditional histological examination using a Leica DM3000 microscope (Leica Microsystems, Wetzlar, Germany) for semi-quantitative assessment of the degree of obesity and inflammation. The examined biopsies contained at least four portal tracts and were informative. The diagnosis of steatosis was made depending on the morphological changes in the hepatic acinus. The diagnosis of NASH was made in presence of hepatic fat infiltration together with lobular inflammation and hepatocyte ballooning. To assess steatosis, a semiquantitative evaluation of morphological changes in the liver was performed by examining digital images of biopsies obtained by scanning histological sections with a Pannoramic 250 FLASH scanning microscope (3DHISTECH, Hungary, Budapest) and Image-J software (ImageJ Wiki, 2021).

### MiRNA PCR Array Analysis

Serum samples were aliquoted into 500 μl each on the collection day and stored at −80°C until later use. According to the manufacturer’s protocol, total RNA, including miRNA, was isolated from 100 μl of serum using MagMAX mirVana Total RNA Isolation Kit (Thermo Fisher Scientific, United States). All isolation steps were performed on a cooling plate. Total RNA was eluted with 50 μl RNAse-free water; the initial concentration range was 8–25 ng/μl. Samples were stored at −70°C until PCR. Before PCR, the quality and fidelity of the RNA were checked. According to the manufacturer’s protocol, Reverse transcription was performed using the miScript II RT Kit (Qiagen, GmbH). 12 μl total RNA, nucleotide mixture and reverse transcriptase were added to HiSpec buffer (Qiagen, GmbH) to a final volume of 20 μl. The mixture was incubated for 60 min at 37°C, and 5 min at 95°C to inactivate miScript II RT Kit reverse transcriptase. 90 μl RNAse-free water was added to each 20 μl of reverse transcription reaction, and 100 μl diluted cDNA was used to make a reaction mix according to the manufacturer’s protocol.

Expression profiling analysis was performed using the miScript miRNA PCR kit (MIHS-106Z, Qiagen, GmbH). The miRNA PCR arrays were performed using a CFX96 (Bio-Rad, Heracles, CA, United States) thermal cycler instrument. The PCR program included the following steps: an initial activation step at 95°C for 15 min, followed by 40 cycles each of denaturation at 94°C for 15 s and annealing at 55°C for 30 s and extension at 70°C for 30 s.

### Statistical Analysis of Experimental Data

The normal distribution of the quantitative indicator was checked using the Shapiro–Wilk test. If the sample fit the normal distribution, the hypothesis of equality of sample means was tested using Student’s *t*-tests. If the data distribution did not obey the law of normal distribution, further assessment of differences between samples was calculated using the non-parametric Mann–Whitney test for pairwise comparisons. Differences were considered significant at *p* < 0.05. Statistical processing of the obtained results was performed using GraphPad Prism 8.01. PCR data were analyzed using miScript miRNA PCR Array Data Analysis Tool software (Qiagen, Hilden, Germany), in which the qRT-PCR modules convert the threshold cycle (Ct) values into the calculated results for miRNA expression and also *via* the comparative Ct method by 2^–ΔΔCt.^ three samples were excluded because they did not pass the quality tests (PCR array reproducibility/RT efficiency:—1 sample from healthy donors and 2 samples from NAFLD patients). To normalize the obtained data, we used the global CT mean of expressed miRNAs (Mestdagh et al., 2009) because small nucleolar RNAs (e.g., SNORD44, SNORD48 and RNU6-1) changed significantly (Marabita et al., 2016; Neagu et al., 2020). All Cq values > 35 were excluded. The most significant differentially expressed miRNAs (>twofold change and *p* < 0.05) in all study groups were selected for subsequent bioinformatics analysis.

### MiRNA Target Mining and Gene Set Enrichment Analysis

MiRNA target genes were predicted using the online platform miRWalk (Sticht et al., 2018) with a binding probability threshold of 100%. MiRWalk uses miRNA sequences from miRBase (version 21). The miRWalk structure includes the TargetScan (version 7.1), miRDB (version 5.0) and miRTarBase (version 7.0) datasets.

Functional Enrichment Analysis (GSEA) was performed in miRWalk using the KEGG (KEGG Pathway Database, 2021) and REACTOME (Home - Reactome Pathway Database, 2021) databases to elucidate the specific biological functions of the resulting gene sets. miRWalk provides a standard enrichment analysis based on hypergeometric tests (chi-square selection algorithm). The resulting GSEA data were analyzed and visualized using R code (version 4.0.2) developed by us in RStudio: Paths were sorted by *p*-values (*p* ≤ 0.05 was considered significantly enriched).

### Comparison of Search Results for Target Genes With NAFLD Pathogenesis

Target genes obtained with the miRWalk platform were compared with a set of genes involved in the pathogenesis of NAFLD. NAFLD pathogenesis genes (158 genes) were exported from the open portal Wiki Pathways [Non-alcoholic fatty liver disease (Homo sapiens)—WikiPathways, 2021]. Data comparisons were performed for the groups: Steatosis, NASH. The NAFLD pathogenesis genes targeted by the miRNAs differentially expressed in our experiment were determined. We analyzed and visualized the obtained data using R code (version 4.0.2) developed by us in RStudio.

## Results

### Liver Biopsy Studies

**FIGURE 2:**
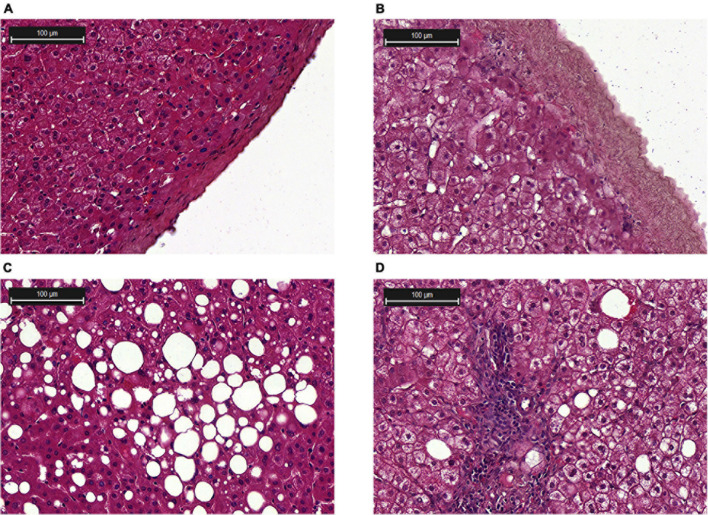
Histological examination of the liver biopsies. **(A)** Liver capsule from a healthy donor; **(B)** Liver capsule with inflammation; **(C)** Steatosis; **(D)** NASH, lymphocytic infiltration in the tissue. Hematoxylin and eosin (H&E) staining.

Histological analysis of liver biopsies showed no signs of inflammation in healthy donors. The lobular organization in biopsies from healthy donors was not altered, and the histoarchitectonics of the hepatic acinus was not changed. The central veins, portal vein, and hepatic tracts were prominent and composed of hepatocytes without signs of polymorphism; only weak manifestations of intracellular cholestasis were noted (Figure 2A).

Morphological changes in liver biopsy at the stages of steatosis and NASH are shown in Figures 2C,D, respectively. The presence of the small, medium and large lipid droplets was characteristic of steatosis. According to the results of histological examination of liver biopsies in the patients with NASH, there was an increase in the area of fatty deposits and the number of lymphocytes (Figure 2D). In the group of patients with NASH, enlarged liver capsule (Figure 2B) and hepatocyte dystrophy were revealed. All patients with NAFLD had an increased area of lipid droplets. The highest levels of lipid infiltration were found in the group of patients with steatosis (Supplementary Figure 1).

### Expression of Circulating miRNAs in Serum of Obese Patients With NAFLD

**FIGURE 3:**
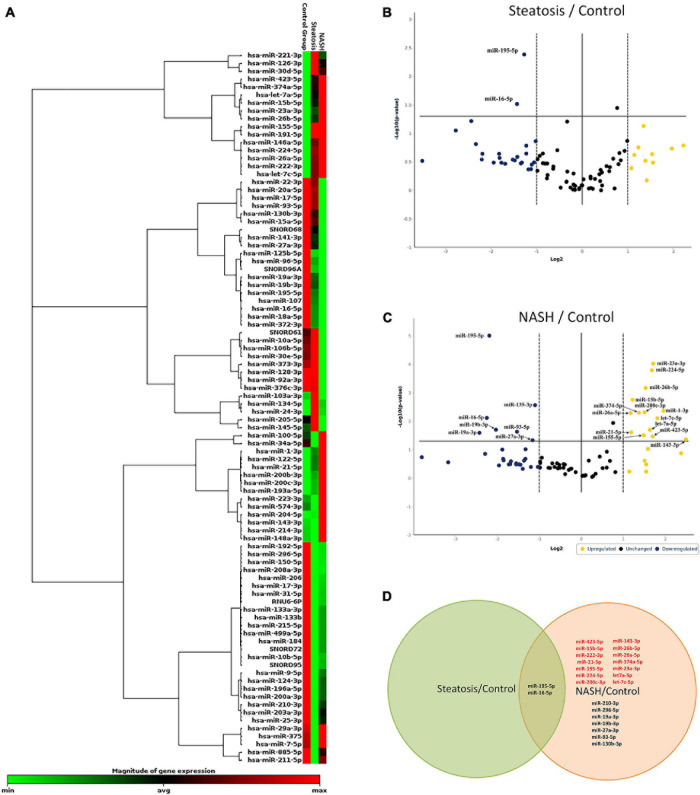
Identification of differentially expressed miRNAs. **(A)** Hierarchical cluster analysis. Red—increased miRNA; green—decreased miRNA; black—no changes in expression. The magnitude of gene expression is determined by calculating the 2-ΔCT for each gene and normalizing to the average 2-ΔCT of all genes across all arrays. Volcano plot of differentially expressed miRNAs in serum; **(B)** group of patients with steatosis and **(C)** group of patients with NASH. Plots show the relationship between fold change and statistical significance. The solid vertical line shows no change in gene expression (log_2_1 = 0). Data points to the right of the solid vertical line indicate activated genes, and data points to the left indicate repressed genes. The dashed lines represent the threshold—2. The solid vertical line represents the threshold of statistical significance, *p* < 0.05. Thus, the blue and yellow dots in the graph indicate >2.0-fold activation and suppression of expression with statistical significance. **(D)** Venn plot of miRNA profile overlap showing common and unique miRNAs in serum from groups of patients with steatosis and NASH. Expression activation is highlighted in red, while statistically significant expression suppression is highlighted in black **(D)**.

We screened 84 miRNAs in serum from NAFLD patients and healthy donors. Patients were divided into groups: healthy donors, groups of patients with steatosis, and NASH. First, unsupervised hierarchical cluster analysis was performed in GeneGlobe Data Analysis Center software to investigate the differential miRNA expression (Figure 3A). According to this, the expression of 51 miRNAs was suppressed, 27 miRNAs were activated, and 6 miRNAs did not change in group patients with steatosis. 49 miRNAs were suppressed in a group patients with NASH, 32 were activated, and 3 did not change.

Volcano plot filtering was performed to identify miRNAs with statistically significant differences in expression in the groups with steatosis and NASH compared with healthy donors. The expression levels of miR-195-5p, miR-16-5p were suppressed (*p* < 0.05) in the group of patients with steatosis (Figure 3B and Supplementary Table 2). The expression levels of miR-23a-3p, miR-224-5p, miR-26b-5p, miR-15b-5p, miR-26a-5p, miR-374a-5p, miR-200c-3p, miR-1-3p, let-7a-5p, miR-21-5p, let-7c-5p, miR-143-3p, miR-423-5p, miR-155-5p were increased (*p* < 0.05), the expression levels of miR- 195-5p, miR-130b-3p, miR-16-5p, miR-19a-3p, miR-19b-3p, miR-27a-3p, miR-93-5p were suppressed (*p* < 0.05) in the group of patients with NASH (Figure 3C and Supplementary Table 3).

Depending on the stage of NAFLD, common and unique miRNAs were found (Figure 3D). For example, 2 miRNAs (miR-195-5p and miR-16-5p) were found in all studied groups. 21 miRNAs were unique to the patients with NASH: miR-222-3p, miR-26b-5p, miR-26a-5p, miR-374-5p, miR-23a-3p, let-7a-5p, miR-155-5p, miR-200c-3p, miR-423-5p, miR-21-5p, miR-224-5p, let-7c-5p, miR-15b-5p, miR-210-3p, miR-296-5p, mir- 19a-3p, mir-19b-3p, miR-27a-3p, miR-93-5p, and miR-130b-3p.

### MiRNA Target Mining and Gene Set Enrichment Assay

**FIGURE 4:**
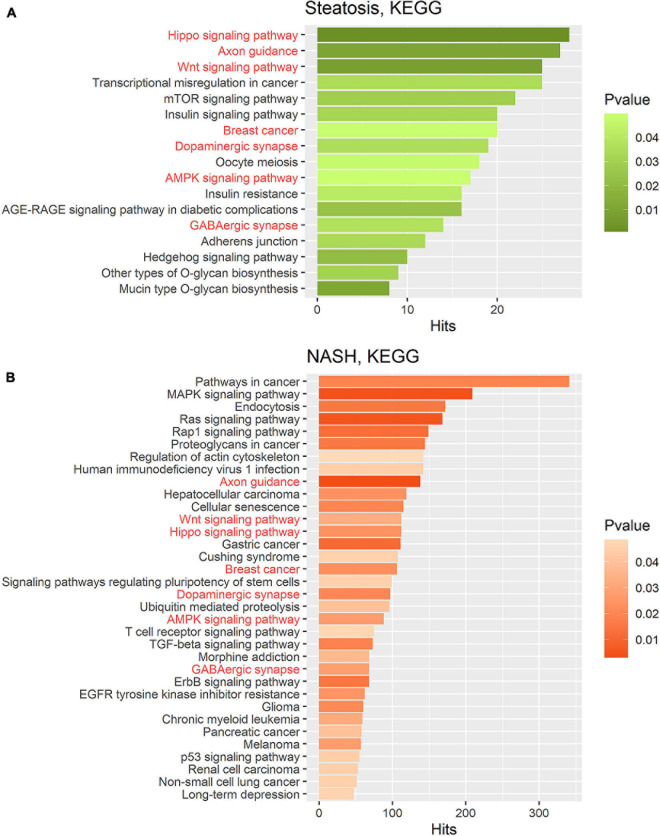
Gene set enrichment analysis performed on the KEGG pathway database for two groups. **(A)** Target gene set of miRNA that were significantly deregulated in the group of patients with steatosis (*p* ≤ 0.05); **(B)** Target gene set of miRNA that were significantly deregulated in the group of patients with NASH (*p* ≤ 0.05). Common pathways in steatosis and NASH are highlighted in red. Hits—number of target genes represented in the pathway.

**FIGURE 5:**
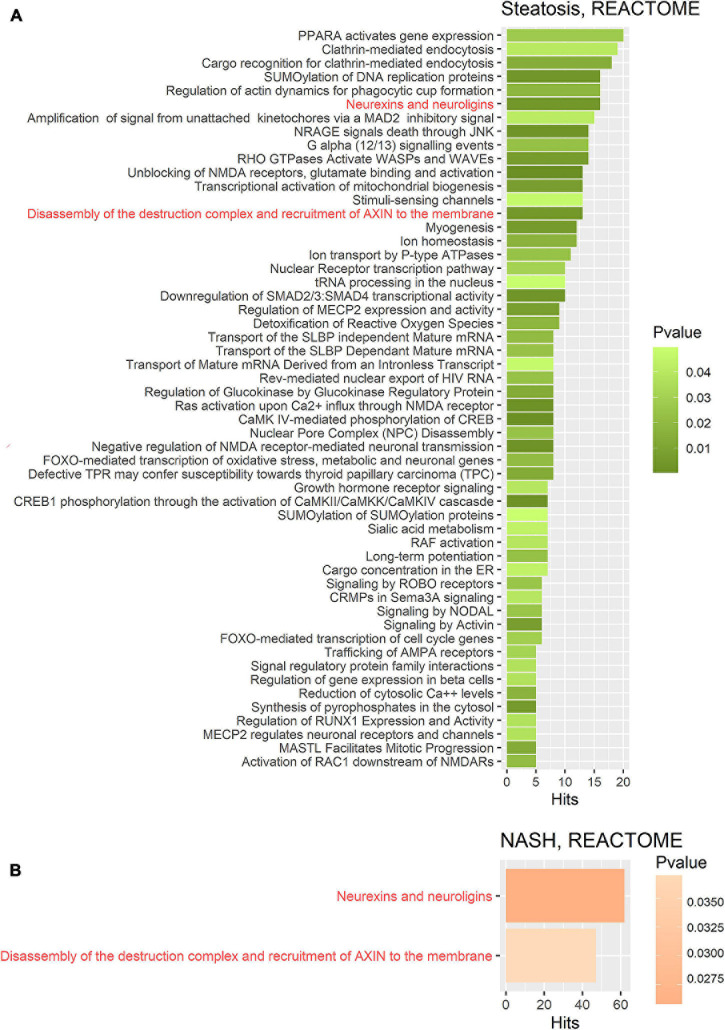
Gene set enrichment analysis performed on the REACTOME metabolic pathway database on two gene sets. **(A)** Target gene set of miRNA that were significantly deregulated in the group of patients with steatosis (*p* ≤ 0.05); **(B)** Target gene set of miRNA that were significantly deregulated in the group of patients with NASH (*p* ≤ 0.05). The common pathways in steatosis and NASH are highlighted in red. Hits—number of target genes represented in the pathway.

To identify the metabolic processes affected by the discovered miRNA deregulation, Gene Set Enrichment Assay (GSEA) was performed using databases and MiRWalk algorithms for two groups: patients with steatosis and NASH. Target genes for differentially expressed miRNAs in referred groups were predicted using the MiRWalk platform. Comparison of GSEA on same gene sets showed that DAVID recognizes fewer genes from similar databases than MiRWalk; therefore, GSEA was performed in MiRWalk. In the group of patients with steatosis, 1 746 putative targets were found for 2 miRNAs. In the group of patients with NASH, 11 106 putative target genes were found for 21 miRNAs. The predicted genes were annotated using the KEGG and REACTOME databases (Figures 4, 5).

KEGG pathway enrichment analysis showed that target genes in the miRNA-mRNA regulatory network were enriched in 7 common pathways in the group of patients with steatosis and NASH: Hippo signaling pathway (KEGG: 04390), Axon guidance (KEGG: 04360), Wnt signaling pathway (KEGG: 04310), Breast cancer (KEGG: 05224), AMPK signaling pathway (KEGG: 04152), Dopaminergic synapse (KEGG: 04728), GABaergic synapse (KEGG: 04727) (Figure 4).

KEGG analysis for the group of patients with steatosis target genes revealed mainly signaling pathways related to insulin signaling and its impairments: mTOR signaling pathways (KEGG: 04150), insulin signaling pathways (KEGG: 04910), insulin resistance (KEGG: 04931), AGE-RAGE signaling pathways in diabetic complications (KEGG: 04933). The most significant number of whole putative target genes were involved in the Hippo signaling pathway (KEGG: 04390) and axon guidance (KEGG: 04360) (more than 25 hits) (Figure 4A).

KEGG analysis for the group of patients with NASH target genes showed that the most represented pathways were those in cancer (KEGG: 05200) (more than 300 hits), indicating the involvement of these miRNAs in the regulation of pathological cell proliferation processes that exacerbate the progression of pathology. In addition, the following pathways were found: MAPK signaling pathway (KEGG: 04010), Endocytosis (KEGG: 04144), RAS signaling pathway (KEGG: 04014), Wnt signaling pathway (KEGG: 04310), Hippo signaling pathway (KEGG: 04390), ErbB signaling pathway (KEGG: 04012), TGF-beta signaling pathway (KEGG: 04350), Cellular senescence (KEGG: 04218) (Figure 4B).

Further GSEA was performed on the REACTOME database (Figures 5A,B). Target gene enrichment analysis in the REACTOME database in the group of patients with steatosis identified 54 metabolic pathways. The largest number of matches (hits) was for the PPAR pathway (PPARA activates gene expression: R-HSA-1989781), endocytosis processes (clathrin-mediated endocytosis: R-HSA-8856828, cargo recognition for clathrin-mediated endocytosis: R-HSA-8856825). In addition, the following significantly enriched pathways were identified: neuro-humoral pathways, mitochondria-dependent energy pathways, pathways associated with carbohydrate metabolism, RNA and protein metabolic pathways, cell death pathways, homeostatic pathways, metabolic pathways, and others.

### Epigenetic Regulation Signatures in NAFLD Pathogenesis in Patients With Steatosis and NASH

**FIGURE 6:**
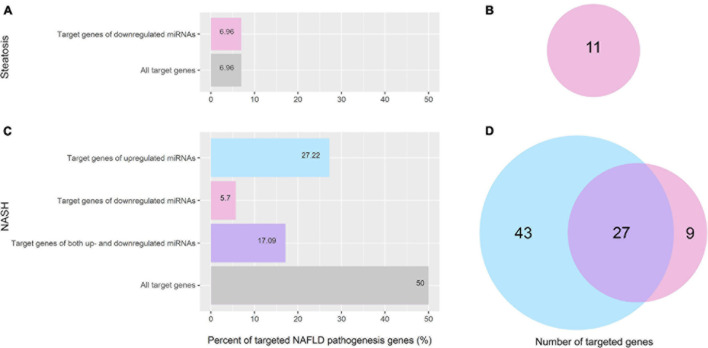
Percentage and amount of NAFLD pathogenesis genes targeted by differentially expressed miRNAs. **(A)** Percentage of NAFLD pathogenesis genes targeted by differentially expressed miRNAs in the group of patients with steatosis; **(B)** Venn plot of NAFLD pathogenesis genes targeted by differentially expressed miRNAs in the group of patients with steatosis; **(C)** Percentage of NAFLD pathogenesis genes targeted by differentially expressed miRNAs in the group of patients with NASH; **(D)** Venn plot of NAFLD pathogenesis genes targeted by differentially expressed miRNAs in the group of patients with NASH. Pink—genes targeted by downregulated miRNAs; light blue—genes targeted by upregulated miRNAs; purple—genes targeted by both up- and downregulated miRNAs.

To uncover the epigenetic mechanisms by which differentially expressed miRNAs contribute to or mitigate the progression of NAFLD, we compared the predicted target genes of differentially expressed miRNAs in the group of patients with steatosis and NASH with genes involved in the NAFLD pathogenesis (158 genes were obtained from the WikiPathways database). The results of this analysis are shown in Figure 6.

In the group of patients with steatosis, the differentially expressed miRNAs target 7% (11 genes) of NAFLD pathogenesis genes. All of them are targeted by downregulated miRNAs (Figures 6A,B). In the group of patients with NASH, miRNAs target 50% (79 genes) of the genes involved in NAFLD pathogenesis (Figures 6C,D). Bioinformatics analysis showed that 43 genes of NAFLD pathogenesis were targeted only by upregulated miRNAs, nine genes were targeted only by downregulated miRNAs, and 27 genes were targeted by both up- and downregulated miRNAs. A complete list of genes targeted by miRNAs for each group is available in the Supplementary Material (Supplementary Tables 3, 4).

## Discussion

Currently, NAFLD has been diagnosed in more than one billion people (Alqahtani et al., 2021). Steatosis may progress to fibrosis and hepatocellular carcinoma or persist for a long time in the form of fatty liver. The reason for the different course of pathogenesis may be due to RNA-dependent epigenetic regulation. Our analysis of circulating miRNAs in the serum of patients with steatosis or NASH showed that disease progression was associated with a significant increase in the number of miRNAs (2 deregulated miRNAs in patients with steatosis versus 21 deregulated miRNAs in patients with NASH). An increase in the number of differentially expressed miRNAs in NASH is associated with pathology progression.

### Deregulation of miR-195-5p and miR-16-5p Is Common in Patients in the Steatosis and NASH Groups

miR-195-5p and miR-16-5p were suppressed in both groups of patients with steatosis and NASH. It is noteworthy that miR-195-5p and miR-16-5p belong to the same miR-15 family and are expressed by cardiomyocytes and fibroblasts (Tijsen et al., 2014). The level of miR-16 in serum correlates positively with NAFLD severity in some studies (Liu X.-L. et al., 2016), negatively in others (López-Riera et al., 2018), or does not correlate at all (Cermelli et al., 2011). miR-16-5p is one of the most commonly used reference standards in microRNA expression assays (Okamoto et al., 2020). Based on the fact that its level changes significantly in the group of patients with NAFLD, the use of miR-16-5p as an internal control needs to be reconsidered in future studies.

### 14 miRNAs Were Upregulated in the Group of Patients With NASH

Analysis of the literature showed that the results of our screening were consistent with other studies: 12 of 14 miRNAs that showed increased expression according to the results of our screening, namely miR-15b, miR-21, miR-23a, miR-26a, miR-155, miR-200c, miR-222, miR-224, miR-374a, miR-423, let-7a and let-7c were upregulated in the serum of patients with NASH in other studies (Cheung et al., 2008; Yamada et al., 2013; Csak et al., 2015; Gerhard and DiStefano, 2015; Pirola et al., 2015; Liu Z. et al., 2016; Dongiovanni et al., 2018; Matsuura et al., 2018; Menghini and Federici, 2018). The levels of miR-23a, miR-224, miR-200, miR-21, miR-423 were increased not only in the serum but also in liver tissue of patients with NASH (Cheung et al., 2008).

Thus, despite the small size of the test groups, our screening results are largely consistent with the results of other studies. We hypothesize that the these miRNAs (miR-15b, miR-21, miR-23a, miR-26a, miR-155, miR-200c, miR-222, miR-224, miR-374a, miR-423, let-7a, and let-7c) might have high potential as diagnostic molecules.

### Pathways of Neurohumoral Regulation Are Epigenetically Modulated in NAFLD

We also aimed to analyze the differences in RNA-dependent epigenetic regulation of signaling cascades and metabolic pathways in the groups of patients with steatosis and NASH. A theoretical understanding of miRNAs’ mechanisms during NAFLD progression was achieved by mining for target genes for differentially expressed miRNAs and then functionally annotating (performing GSEA analyzes) the predicted genes using the KEGG and REACTOME databases.

Gene set enrichment analysis on the KEGG database for the patients with steatosis revealed pathways associated with cell cycle regulation, regeneration, apoptosis, and insulin signaling. Pathways were characterized by the number of hits (the number of genes targeted by differentially expressed miRNAs) and the significance of enrichment (*p* < 0.05). The Hippo signaling pathway plays a crucial role in the processes of apoptosis, proliferation, and cell regeneration (Nguyen-Lefebvre et al., 2021). The Hippo signaling pathway is an essential modulator of liver metabolism (Koo and Guan, 2018), acting through the hormone receptor GPCR. One of the types of post-translational protein modifications is glycosylation. The mucin-type O-glycan biosynthesis pathway is responsible for glycosylation and protects the cell surface from external stress, microbial infection, and autoimmune responses (Reily et al., 2019). *O*-glycans play an important role in lipid metabolism: deficiency of O-glycans during embryonic and postnatal development disrupts lipid transport and leads to fatty liver (Fu et al., 2008). Thus, signal transduction pathways and post-translational modification (glycosylation) of proteins were the most enriched pathways.

For a more detailed characterization, a GSEA was performed on the REACTOME database for the group of patients with steatosis. The GSEA based on REACTOME resulted in a larger number of enriched pathways due to the lower hierarchy of the REACTOME database. The analysis for the group of patients with steatosis showed that more than ten represented pathways were involved in neuro-humoral regulation. The most enriched pathway for the group of patients with NASH was the neurexins and neuroligins pathway. Neurexins and neuroligins are synaptic adhesion molecules in the central nervous system that regulate the differentiation and maturation of glutamatergic (excitatory) or GABAergic (inhibitory) synapses (Craig and Kang, 2007). Neuroligin family members have been found to stimulate insulin production, demonstrating the importance of neurohumoral regulation in steatosis (Suckow et al., 2008). Dopaminergic pathways play an important role in the regulation of appetite and eating behaviors, as well as inflammation associated with obesity (Leite and Ribeiro, 2020). Dopaminergic pathways are found in many tissues and can influence glucose homeostasis and body weight (Matt and Gaskill, 2020). Activation of the intrahepatic GABAergic system protects the liver from damage (Wang et al., 2017). Signaling pathways involved in neurohumoral regulation are strongly represented in the group of patients with steatosis. These findings provide a foundation for further research and understanding of how these pathways of RNA-dependent epigenetic regulation are involved in the pathogenesis of NAFLD and the impact this may have on disease progression.

Signaling pathways associated with synapse formation and plasticity (axon guidance, dopaminergic synapse, and GABAergic synapse) were common in steatosis and NASH. This finding reveals new neurohumoral aspects of epigenetic regulation of metabolic diseases that can be considered in future studies.

In contrast to the group of patients with steatosis, GSEA on the KEGG database for the group of patients with NASH revealed multiple pathways associated with cancer (approximately ten pathways). The enriched pathways highlight the potential of NASH to develop into malignant transformation (pathological proliferation). Moreover, the mechanisms of epigenetic regulation associated with NAFLD progression based on KEGG are associated with the most significant pathways: regulation of cell cycle, proliferation, differentiation and migration of cells, cellular and humoral responses. However, the most enriched Reactome pathway for the NASH group was the neurexins and neuroligins pathway.

### Differentially Expressed miRNAs Target Genes Responsible for Oxidative Phosphorylation (Mitochondrial Function) in Groups of Patients With Steatosis and Steatohepatitis

To identify specific miRNA-mRNA interactions within NAFLD, we focused on signaling pathways of NAFLD pathogenesis exported from Wiki Pathways. We found that miRNAs deregulated according to our screening target 50% of NAFLD pathogenesis genes in the group of patients with NASH and 7% of NAFLD pathogenesis genes in the group of patients with steatosis. RNA-dependent epigenetic regulation is enhanced in correlation with pathology progression.

**FIGURE 7:**
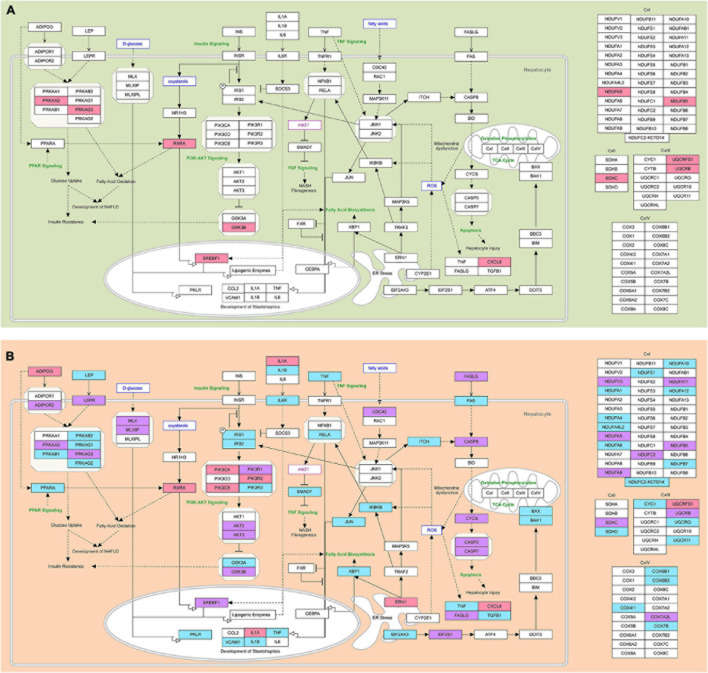
The modified NAFLD pathogenesis map showing epigenetic regulatory signatures in steatosis and NASH. **(A)** Predicted NAFLD pathogenesis genes targeted by differentially expressed miRNAs in the group of patients with steatosis are highlighted; **(B)** Predicted NAFLD pathogenesis genes targeted by differentially expressed miRNAs in the group of patients with NASH are highlighted. Pink—genes targeted by downregulated miRNAs; light blue—genes targeted by upregulated miRNAs; purple—genes targeted by both up- and downregulated miRNAs. The pathway was imported from WikiPathways.

In the group of patients with steatosis, the target genes of decreased miR-16-5p and miR-195-5p are involved in Electron Transport Chain (OXPHOS system in mitochondria): Complex I—NDUFA5, NDUFB5, Complex II—SDHC, Complex III—UQCRB, UQCRFS1, lipid metabolism (genes: PRKAA2, PRKAG3, RXRA, SREBF1, GSK3B) and chemokine signaling (CXCL8) (Figure 7A and Supplementary Table 3). An increase in the activity of these genes due to the downregulation of miR-16-5p and miR-195-5p could be an adaptive mechanism to restrain the progression of steatosis.

Mitochondria are able to adapt rapidly to external signals in order to maintain cell homeostasis and metabolic activity. They play a key role in energy production and cell death control (Longo et al., 2021). In obesity, the development of oxidative stress leads to the formation of peroxynitrite, which causes damage to the mitochondrial electron transport chain *via* cytochrome C and increases the production of reactive oxygen species (ROS). Oxidative stress promotes damage to mtDNA, leading to mitochondrial dysfunction (Skuratovskaia et al., 2020). In previous work, we have shown that mitochondrial dysfunction may contribute to the development of type 2 diabetes in obese patients with an inflammatory process in the liver (Skuratovskaia et al., 2021). This work emphasized the effect of increased expression of miRNAs in patients with NASH on the repression of genes for mitochondrial biogenesis.

In the group of patients with NASH, miRNAs were found to target genes largely responsible for the functioning of mitochondria, which are abundant in the liver (Degli Esposti et al., 2012). Our data show that the upregulated miRNAs targeted genes of mitochondrial complex I (NADH: ubiquinone oxidoreductase), complex II (succinate dehydrogenase), complex III (cytochrome bc1 complex), and complex IV (cytochrome c oxidase) (Figure 7B and Supplementary Table 4). In the group of patients with NASH, miRNAs likely decrease ATP synthesis by altering the expression of OXPHOS subunits. Probably, genes responsible for ATP production were suppressed by increased miRNAs (let-7c-5p, miR-155-5p, miR-423-5p, miR-15b-5p, miR-143-3p, and miR-26a-5p) in the group of patients with NASH compared to the steatosis group.

There are studies that predict microRNAs from a targeted sequence. Thus, the authors found polymorphic variants of genes in the mitochondrial D-loop (Hasturk et al., 2019). Mitochondrial DNA variants m.16129 G > A, AA genotype and m.16249 T > C, CC genotype, were associated with fibrosis and lobular inflammation and histological steatosis in NASH patients, respectively (Hasturk et al., 2019). They predicted several miRNAs for each polymorphic gene variant in target sequences. The authors investigated the possible correlation of the mtDNA copy number with the rs3746444 MIR499A polymorphism in another study. It was shown that the homozygous variant genotype for the rs3746444 MIR499A polymorphism correlates with a decrease in the number of mtDNA copies, especially in patients with T2DM (*p* = 0.009). The association of the variant MIR499A allele with the mtDNA copy number suggests a possible effect of this polymorphism on oxidative stress (Latini et al., 2020).

### Differentially Expressed miRNAs Alter Energy Balance in the Liver by Targeting Genes of the AMPK Complex

AMPK is a central regulator of cellular metabolism that is activated by a decrease in intracellular ATP levels. AMPK signaling reduces lipogenesis by inhibiting acetyl CoA carboxylase (ACC). The AMPK complex consists of a catalytic subunit (α) and two regulatory subunits (β and γ) (Mihaylova and Shaw, 2011). AMPK activity in the liver is reduced in patients with NAFLD in the presence of excess nutrients. In our work, the upregulated miRNAs target the regulatory subunit genes of the AMPK complex: let-7a-5p, let-7c-5p, miR-26a-5p, miR-423-5p target PRKAB1; let-7c-5p, mir-423-5p target PRKAG1; let-7c-5p, mir 15b-5p target PRKAG2; miR-15b-5p—PRKAB2 (Figure 7B and Supplementary Table 4). Suppression of B-subunit gene expression disrupts the formation of the complex required for AMPK interaction with downstream targets. The binding of ADP and AMP to the G-subunit of AMPK triggers phosphorylation of Thr172 and maintains the kinase in an active conformation (Willows et al., 2017). Suppression of G-subunit gene expression can suppress the activity of the AMPK complex. In the work of Simino et al. (2021), Prkaa2 (AMPKα2) was shown to be a direct let-7 target in the liver in a steatosis model (Simino et al., 2021). In general, a decrease in the expression of AMPK complex genes by RNA-dependent epigenetic regulation indicates dysfunction of the complex, consistent with data from other researchers. Let-7a-5p, let-7c-5p, miR-15b-5p, miR-26a-5p, miR-423-5p, inhibit the AMPK complex, disturbing the energy balance in the liver, contributing to the formation of NASH.

Leptin (LEP) and adiponectin (ADIPOQ), hormones of adipose tissue (adipokines), can activate AMPK. In obesity, the production of leptin increases, and the secretion of adiponectin is suppressed. Dysfunction of adipokines is associated with the progression of NAFLD. Upregulated miR-200c-3p and miR-224-5p target the gene LEP and lead to its suppression (Figure 7B and Supplementary Table 4). Hyperleptinemia has been shown to lead to the development of insulin resistance (the pathways of leptin and insulin cross), inhibition of lipid oxidation, and activation of lipogenesis by SREBP-1 in the liver (Jiménez-Cortegana et al., 2021). Leptin can activate HSC, provoking the development of fibrosis (Polyzos et al., 2011). Therefore, decreasing LEP gene expression through RNA-dependent epigenetic regulation is a promising therapeutic mechanism as it leads to a decrease in lipid production in the liver.

AMPK is closely linked to mitochondrial biogenesis *via* PPAR. PPAR forms a heterodimer with RXR, and this complex transactivates target genes to adapt cells to metabolic changes (Lee and Kim, 2010). The transcription factor PPAR plays a vital role in lipid metabolism. Thus, deletion of Pparα in mouse liver contributes to steatosis and NASH in obesity (Régnier et al., 2020). The increase in miR-423-5p suppresses the expression of the PPARA gene, which may lead to a decrease in fatty acid oxidation and an increase in lipogenesis in the liver.

### Therapeutic Increase of miR-423-5p Can Decrease *de novo* Lipogenesis in the Liver

Another potential target of mir-423-5p is the PKLR gene (Figure 7B and Supplementary Table 4). Elevated L/R-type pyruvate kinase (PKLR) levels are associated with NAFLD progression: thus, inhibition of PKLR lowers liver triglyceride and cholesterol levels and improves insulin sensitivity in mice models (Krishnan et al., 2018). In addition, it has been shown that inhibition of PKLR in the liver leads to a decrease in OXPHOS in steatosis, suppressing mitochondrial respiration and reducing active production of ROS, which alleviates fatty liver. However, in fibrosis, PKLR inhibition has the opposite effect (Krishnan et al., 2018). In light of this, suppression of PKLR by mir-423-5p to reduce *de novo* lipogenesis and improve mitochondrial function may have potential as a therapy in the early stages of NAFLD.

### Differentially Expressed miRNAs Target Genes Responsible for Mechanisms of Cell Death

Dysregulation of enzymes (I-III complexes) and mitochondrial biogenesis leads to the formation of reactive oxygen species (ROS) that trigger cell death (Duarte et al., 2014; Redza-Dutordoir and Averill-Bates, 2016). Cells die by necrosis if there is not enough ATP or apoptosis if there is enough ATP (Duarte et al., 2014).

The extrinsic pathway of apoptosis is triggered *via* death receptors (DR) or pattern recognition receptors (PRR) (Galluzzi et al., 2018). DR includes the Fas cell surface death receptor (FAS; also known as CD95 or APO-1) and a member of the TNFR superfamily of receptors (Brenner et al., 2015). Elevated miR-143-3p targets the gene FAS according to bioinformatics prediction (Figure 7B and Supplementary Table 4). FAS (CD95, TNFRSF6) induces assembly of the death-inducing signaling complex (DISC), leading to apoptosis (Lavrik and Krammer, 2012). The upregulated miR-423-5p targets the TNF gene. We hypothesize that elevation of miR-143-3p and miR-423-5p, aimed at suppressing the expression of FAS and TNF genes, may prevent apoptotic signaling and programmed cell death, contributing to the development of fibrosis and hepatocarcinogenesis.

Interestingly, miR-423-5p targets the downstream genes of the TNF/NF-kB signaling pathway: RelA, JUN, IKBKB (Figure 7B and Supplementary Table 4). NF-κB is the major transcriptional pathway that protects hepatocytes from TNF- and LPS-induced cell death (Luedde and Schwabe, 2011). Normal NF-κB function is required to synthesize cytokines (TNF, IL -6) and control hepatocyte proliferation after exposure to oxidative stress (Ringelhan et al., 2012). Blocking the RelA subunit disrupts NF-kB activation and renders hepatocytes vulnerable to the lethal effects of TNF (Geisler et al., 2007). IKK-beta-deficient hepatocytes counter with weak NF-κB activation in response to TNF in animal models. RelA deficiency was associated with prolonged JNK activation and elimination of the anti-apoptotic cellular protein inhibitor FLICE (c- FLIP) (Geisler et al., 2007). Inhibition of JUN (p39; AP -1; c-Jun) promotes hepatocyte death in a background of oxidative stress and compensatory regeneration. Suppression of c-Jun and NF-κB promotes hepatocyte death and impedes liver tissue regeneration (Hasselblatt et al., 2007; Le Clorennec et al., 2019). MiR-423-5p also acts on IKBKB (an inhibitor of the beta subunit of nuclear factor kappa B kinase). IKK phosphorylates NF-kB inhibitors and stimulates their degradation, allowing free NF-kB to migrate into the nucleus and activate the transcription of hundreds of genes involved in immune response, growth control, or protection against apoptosis. In addition to NF-kB inhibitors, IKK phosphorylates several other pathway components, including the NF-kB subunits RELA and NFKB1 and IKK-linked kinases (Salmerón et al., 2001; Yoboua et al., 2010). Reduction of the activity of IKB and RelA and JUN by miR-423-5p may lead to inhibition of the NF-kB pathway and inactivation of pro-apoptotic factors, contributing to the progression of NASH.

Let-7c-5p and miR-423-5p target genes associated with the mitochondrial (intrinsic) pathway of apoptosis, namely the apoptosis activation genes BAK1 and BAX (Figure 7B and Supplementary Table 4). BAK1 is a member of the BCL2 protein family that functions as a pro-apoptotic regulator. BAK and BAX form homo-oligomers within the mitochondrial membrane, resulting in the release of cytochrome C, which activates Apaf1 and leads to the activation of caspase 9 (Nguyen et al., 2018). Let-7c-5p has a neuroprotective effect by inhibiting the expression of pro-caspase-3 and caspase-3 and preventing cell apoptosis after stroke (Tao et al., 2015). Overexpression of let-7c-5p inhibited EtOH-induced hepatic steatosis and apoptosis (Wang et al., 2018). Thus, let-7c-5p and miR-423-5p can suppress hepatocyte apoptosis in NAFLD.

Other miRNAs, miR-143-5p and miR-200c-3p, which are upregulated in patients with NASH, target the ITCH gene (Figure 7B and Supplementary Table 4). ITCH is a HECT ubiquitin ligase E3 that controls programmed cell death through ubiquitin-induced degradation of anti-apoptotic proteins (Chang et al., 2006). In contrast, suppression of the ITCH gene by miR-143-5p and miR-200c-3 can promote apoptosis. No signs of malignant tissue degeneration were found in patients included in the study, likely due to compensatory maintenance of apoptotic processes in response to upregulation of miR-143-5p and miR-200c-3. We anticipate that future experimental studies aimed at understanding the role of miR-423-5p, miR-143-5p, and miR-200c-3 in regulating apoptotic processes in liver and hepatocarcinoma will be of great importance.

### Differentially Expressed miRNAs Target Genes Involved in the Unfolded Protein Response Pathway

Oxidative stress in fatty liver disease triggers ER stress, which promotes the formation of misfolded proteins and NASH progression. According to the performed GSEA, the signaling pathways and processes associated with post-translational protein modification were predicted to be the most important in the group of patients with NASH. The development of endoplasmic reticulum stress is associated with activating UPR-pathway, which controls protein homeostasis and lipid metabolism (Lebeaupin et al., 2018). The PERK pathway is one of the three major signaling branches of the UPR (Hetz, 2012). The increase in mir-26a-5p was targeted to the EIF2AK3 (PERK) gene (Figure 7B and Supplementary Table 4). PERK-dependent ATF4 activation induces the expression of stress-sensitive genes responsible for cellular redox potential and apoptosis signaling (Romine and Wiseman, 2019). Upregulated mir-26a-5p targets the gene PERK and may aim to suppress antioxidant protection and activate apoptosis. Another signaling branch of the UPR pathway is ATF6. ATF6α activates transcription of XBP1 (Yoshida et al., 2001). The upregulated mir-423-5p and let-7a-5p target the XBP1 gene. In hepatocytes, XBP1 regulates liver lipogenesis by directly binding to promoters of lipogenic genes (SCD1, DGAT2, and ACC2) and activating their expression. It was shown that *de novo* lipid biosynthesis was reduced in the liver of mice with deletion of XBP1 (Zhang et al., 2014). Thus, a decrease in XBP1 gene expression by mir-423-5p and let-7a-5p could result in a suppression of lipid biosynthesis.

### Differentially Expressed miRNAs Target IL1B, IL6, IL6R, and VCAM1

According to the results of bioinformatic prediction of genes targeted by differentially expressed miRNAs, it was found that the processes of ATP synthesis and apoptosis were reduced, and oxidative stress and inflammation were increased in the group of patients with NASH. Hepatocyte necrosis is considered an unprogrammed form of cell death resulting from metabolic failure (Hotchkiss et al., 2009). Many authors associate necrosis with a significant inflammatory response triggered by releasing the pro-inflammatory cytokines IL-1, TNF (Kany et al., 2019). IL-1α directly or *via* induction of pro-inflammatory cytokines (IL-6, TNFα, and IL-1β) promotes steatohepatitis by increasing the expression of adhesion molecules and chemotactic factors. IL-1β stimulates the accumulation of triglycerides and cholesterol in primary hepatocytes and the formation of lipid droplets in the liver (Negrin et al., 2014). The upregulated let-7c-5p targets the IL1B gene (group of patients with NASH). In several cellular models, it has been shown that an increase in IL-1β production leads to the progression of inflammation and activation of HSC (Miura et al., 2010). Thus, suppression of IL-1β expression by increasing let-7c-5p may lead to a reduction in NASH progression, mediated by a decrease in lipogenic enzyme activity, a decrease in migration of immunocompetent cells, and suppression of metalloproteinase production.

IL-1β stimulates the release of IL-6. IL-6 binds to IL-6Ra or IL-6Rb and initiates signaling (Rose-John, 2018). Classical IL-6 signaling occurs *via* IL-6Ra, stimulates liver regeneration, and protects the liver from damage (Skuratovskaia et al., 2021). IL-6R is downregulated by upregulated miR-423-5p, let-7a-5p, let-7c-5p in the group of patients with NASH. Sui et al. (2019) showed that IL-6R was identified as a direct target of let-7a (Sui et al., 2019). The authors showed that the let-7a inhibitor significantly increased the level of IL-6R expression, resulting in increased proliferation of liver cells. IL-6 is involved in the regulation of various cellular functions. Inhibition of miR-423-5p, let-7a-5p, let-7c-5p can restore the classical pathway of IL-6 signaling in the liver.

TNF-α and IL -1β, LPS, and ROS can induce the expression of VCAM-1 through different signaling pathways (NF-κB, IRF-1, and Ap-1) (Furuta et al., 2021). VCAM-1 is a cell adhesion protein that regulates leukocyte adhesion to vascular endothelium (Carr, 2021). VCAM expression by liver sinusoidal endothelial cells is significantly increased in NASH patients. The upregulated let-7c-5p is predicted to target the VCAM1 gene. Pharmacological inhibition or genetic deletion of VCAM-1 in liver endothelial cells has been shown to reduce liver inflammation, injury, and fibrosis in NASH mice (Furuta et al., 2021) 2021). Suppression of VCAM1 expression by let-7c-5p may be protective against the development of NASH.

### Differentially Expressed miRNAs Target Genes Involved in TGF Signaling

According to target mining, the upregulated miRNA-423-5p targets the TGFB1 gene. Signaling of the transforming growth factor TGF-β activates Smad-dependent and TAK1-dependent signaling pathways necessary to regulate proliferation, cell survival, fibrosis, and tumorigenesis (Yang et al., 2014). The activation of the TGF-β pathway in the liver is associated with hepatocyte damage, infiltration of inflammatory cells and their production of inflammatory cytokines, activation of HSC, and development of fibrosis. Therefore, suppression of genes associated with activation of this pathway will reduce the progression of NAFLD. Another component of the TGF-β signaling pathway is the SMAD7 gene. An increase in miRNA-423-5p, miR-155-5p and mir-200c-3p can suppress the SMAD7 gene. Thus, SMAD7 inhibits TGF-β signaling by acting downstream of the TGF-β receptor (Stopa et al., 2000). Smad7 deficiency contributes to the development of alcoholic fatty liver disease (Zhu et al., 2011). Song et al. (2020) showed that the miR-200c-3p-SMAD7 regulatory axis promotes bone tissue formation (Song et al., 2020). On the one hand, an increase in miRNA-423-5p reduces the expression of the TGFB1 gene; on the other hand, miRNA-423-5p, miR-155-5p, and mir-200c-3p reduce the expression of the TGF-β pathway inhibitor, SMAD7 gene. Therefore, further research is needed to understand the role of miRNA-423-5p in the regulation of the TGF-β signaling pathway.

### Differentially Expressed miRNAs Target Genes Involved in Insulin and PI3K-AKT Signaling

Differentially expressed microRNAs target genes that are also involved in insulin signaling. For example, the upregulated miRNA-143-3p and miR-423-5p target insulin receptor substrates 1 and 2 (IRS1 and 2), respectively (Figure 7B and Supplementary Table 4). The miRNA-143-3p—IRS1 relationship was experimentally confirmed: deletion of miRNA-143 in vascular smooth muscle cells leads to a substantial increase in IRS1 expression and insulin signaling, and thus insulin-induced glucose uptake in smooth muscle cells. It has also been shown that suppression of IRS1 activity can contribute to necroinflammatory liver activity (Lan and Albinsson, 2020). An increase in miRNA-143-3p and subsequent suppression of IRS1 may lead to the formation of NASH *via* the development of insulin resistance and hyperglycemia in the liver.

According to the target mining results, many phosphoinositide 3-kinase (PI3K) family genes underwent epigenetic regulation by deregulated miRNAs. Upregulated miRNA-423-5p and miR-21-5p target the PIK3R3 gene, which has phosphatidylinositol kinase activity and can activate IRS1. Activation of PIK3R3 promotes the oxidation of fatty acids in the liver *via* PPARα. Thus, a decrease in the expression of PIK3R3 by an increase in miRNA-423-5p and miR-21-5p may result in suppression of the insulin signaling pathway and activation of lipid accumulation processes.

Upregulated miR-423-5p targets glycogen synthase kinase-3 alpha (GSK3A) (Figure 7B and Supplementary Table 4). Inhibition of GSK3 leads to dephosphorylation and activation of glycogen synthase and maybe a liver defense mechanism. GSK3 inhibitors have been considered as agents for targeted therapy for insulin resistance since the 1970s (Maqbool and Hoda, 2017). Thus, the increase in miR-423-5p through an inhibitory effect on GSK3 has therapeutic potential for the treatment of insulin resistance in the context of NAFLD. Of note, inhibition of GSK3 also promotes liver regeneration in the presence of toxic damage (Bhushan et al., 2017).

In summary, let-7c-5p, miR-15b-5p, miR-21-5p, miR-423-5p, and miR-143-3p are of particular interest for further research and potential therapy of insulin resistance. The latter two have been little studied in NAFLD; there is no experimental evidence for targeting the predicted genes. Of note is the ambivalence of the effects of miR-423-5p and miR-143-3p: increasing their expression is fruitful from the point of view of possible suppression of GSK3. On the other hand, this leads to an inhibition of IRS1, which stimulates the development of NASH.

## Conclusion

Thus, miR-15b, miR-21, miR-23a, miR-26a, miR-155, miR-200c, miR-222, miR-224, miR-374a, miR-423, let-7a, and let-7c have great potential as diagnostic biomarkers of NASH in serum. Our study indicates the need to reconsider the use of miR-16-5p as an internal control in future studies. The major signaling pathways involved in the progression of NASH were signal transduction pathways, post-translational modification (glycosylation) of proteins, pathways involved in neurohumoral regulation, and pathways associated with the development of malignant cell transformation. The data we obtained as a result of bioinformatic analysis require confirmation by experimental studies. We found that miR-374a-5p, miR-1-3p, and miR-23a-3p do not target genes directly involved in the pathogenesis of NAFLD. The study of the obtained data will reveal new regulatory relationships involved in the pathogenesis of NASH.

Suppression of the processes of apoptosis in the liver promotes necrosis, fibrosis, and hepatocarcinogenesis. No signs of malignant tissue degeneration were found in the patients included in the study, probably due to the compensatory maintenance of apoptotic processes realized by miR-143-5p and miR-200c-3. The role of miRNAs miR-423-5p, miR-143-5p, and miR-200c-3 in regulating apoptotic processes in liver and hepatocarcinogenesis in humans is interesting for future experimental studies. According to bioinformatic predictions, ATP synthesis and apoptosis processes were reduced, and oxidative stress and inflammation were increased in the group of patients with NASH.

Inhibition of miR-423-5p, let-7a-5p, let-7c-5p can restore the classical type of IL -6 signaling in the liver. Upregulated miRNA-423-5p reduces the expression of the TGFB1 gene on the one hand; on the other hand, miRNA-423-5p, miR-155-5p, and mir-200c-3p reduce the expression of the TGF-β pathway inhibitor SMAD7 gene. Therefore, further studies are needed to understand the role of miRNA-423-5p in the regulation of the TGF-β signaling pathway. In summary, let-7c-5p, miR-15b-5p, miR-21-5p, miR-423-5p and miR-143-3p are of particular interest for further research and potential therapy of insulin resistance. The latter two have been poorly studied in the context of NAFLD; there is no experimental evidence of targeting the predicted genes. These findings provide the basis for further research and understanding of how these pathways of RNA-dependent epigenetic regulation are involved in the pathogenesis of NAFLD and the impact this may have on disease progression.

## Data Availability Statement

The original contributions presented in the study are included in the article/Supplementary Material, further inquiries can be directed to the corresponding author/s.

## Ethics Statement

The studies involving human participants were reviewed and approved by Local Ethics Committee of the Baltic Federal University I. Kant, Protocol No. 4 of November 29, 2018. The patients/participants provided their written informed consent to participate in this study.

## Author Contributions

MV: conceptualization and formal analysis. LL and EK: validation. MV and DS: writing—original draft preparation. MV, DS, LL, and IK: writing—review and editing. MB: bioinformatics analysis. MV and MB: visualization. MV, AK, and NG: experimental work. IK: advice on the design of the clinical data and experimental material in this study. All authors contributed to the article and approved the submitted version.

## Conflict of Interest

The authors declare that the research was conducted in the absence of any commercial or financial relationships that could be construed as a potential conflict of interest.

## Publisher’s Note

All claims expressed in this article are solely those of the authors and do not necessarily represent those of their affiliated organizations, or those of the publisher, the editors and the reviewers. Any product that may be evaluated in this article, or claim that may be made by its manufacturer, is not guaranteed or endorsed by the publisher.
