# Supplementary material for: Analysis of miRNAs Profiles in Serum of Patients With Steatosis and Steatohepatitis
Source: Front Cell Dev Biol. 2021 Sep 9;9:736677. doi: 10.3389/fcell.2021.736677 (PMC8458751; doi:10.3389/fcell.2021.736677)
Supplement: Supplementary file 1 [file Data_Sheet_1.docx]

Supplementary Material


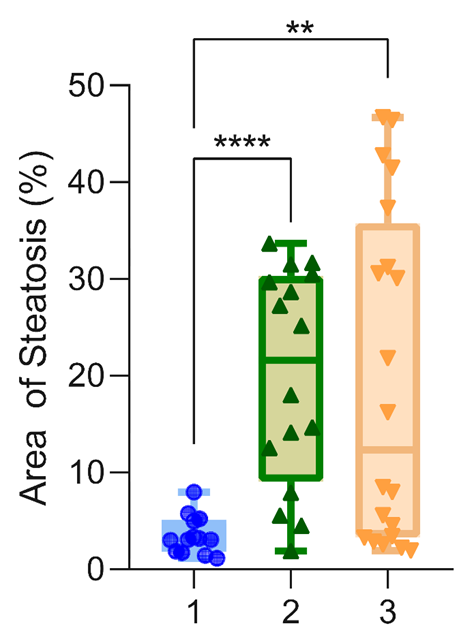


**Figure 1.** The area of lipid droplets in histological analysis.

Note: 1 - **Healthy donors**, 2 - Group of patients with steatosis, 3 - Group of patients with NASH.

| **№** | **miRNA** | **P-value** | **Fold change** |
| --- | --- | --- | --- |
| **1** | miR-195-5p | 0,0041 | -2.41 |
| **2** | miR-16-5p | 0,0305 | -2.69 |

**Supplementary Table 1.** Differential expression miRNAs in serum of group of patients with steatosis.

| **№** | **miRNA** | **P-value** | **Fold change** |
| --- | --- | --- | --- |
| **1** | miR-26b-5p | 0.0006 | 2.89 |
| **2** | miR-26a-5p | 0.0051 | 2.26 |
| **3** | miR-374a-5p | 0.0050 | 2.59 |
| **4** | let-7a-5p | 0.0078 | 3.52 |
| **5** | miR-1-3p | 0.0020 | 4.06 |
| **6** | miR-143-3p | 0.0432 | 5.63 |
| **7** | miR-155-5p | 0.0158 | 2.92 |
| **8** | miR-200c-3p | 0.0019 | 2.97 |
| **9** | miR-21-5p | 0.0248 | 2.28 |
| **10** | miR-224-5p | 0.0002 | 3.34 |
| **11** | miR-23a-3p | 0.0000 | 3.28 |
| **12** | miR-423-5p | 0.0343 | 3.25 |
| **13** | let-7c-5p | 0.0200 | 3.11 |
| **14** | miR-15b-5p | 0.0017 | 2.33 |
| **15** | miR-195-5p | 0.0000 | -4.56 |
| **16** | miR-19a-3p | 0.0256 | -5.37 |
| **17** | miR-19b-3p | 0.0198 | -4.09 |
| **18** | miR-27a-3p | 0.0468 | -2.23 |
| **19** | miR-93-5p | 0.0234 | -2.88 |
| **20** | miR-130b-3p | 0.0027 | -2.14 |
| **21** | miR-16-5p | 0.0076 | -4.75 |

**Supplementary Table 2.** Differential expression miRNAs in serum of group of patients with NASH.

|  | **Genes targeted by downregulated miRNAs** | **Downregulated miRNAs** |
| --- | --- | --- |
| 1 | CXCL8 | hsa-miR-195-5p |
| 2 | GSK3B | hsa-miR-195-5p |
| 3 | NDUFA5 | hsa-miR-195-5p |
| 4 | NDUFB5 | hsa-miR-16-5p, hsa-miR-195-5p |
| 5 | PRKAA2 | hsa-miR-16-5p |
| 6 | PRKAG3 | hsa-miR-16-5p |
| 7 | RXRA | hsa-miR-16-5p |
| 8 | SDHC | hsa-miR-195-5p |
| 9 | SREBF1 | hsa-miR-16-5p |
| 10 | UQCRB | hsa-miR-195-5p |
| 11 | UQCRFS1 | hsa-miR-195-5p |

**Supplementary Table 3.** NAFLD pathogenesis genes targeted by differentially expressed microRNAs in group of patients with steatosis.

|  | **Genes targeted by upregulated miRNAs** | **Targeting upregulated miRNAs** |  | **Genes targeted by downregulated miRNAs** | **Targeting downregulated miRNAs** |  | **Genes targeted by modulated miRNAs** | **Targeting both up- and downregulated  miRNAs** |
| --- | --- | --- | --- | --- | --- | --- | --- | --- |
| 1 | BAK1 | hsa-let-7c-5p |  | ADIPOQ | hsa-miR-130b-3p |  | ADIPOR2 | hsa-let-7a-5p, hsa-let-7c-5p, hsa-miR-130b-3p, hsa-miR-15b-5p |
| 2 | BAX | hsa-let-7c-5p, hsa-miR-423-5p |  | CXCL8 | hsa-miR-195-5p |  | AKT2 | hsa-let-7a-5p, hsa-miR-15b-5p, hsa-miR-27a-3p |
| 3 | COX4I1 | hsa-let-7c-5p |  | ERN1 | hsa-miR-130b-3p |  | AKT3 | hsa-let-7a-5p, hsa-let-7c-5p, hsa-miR-130b-3p, hsa-miR-15b-5p, hsa-miR-19b-3p, hsa-miR-423-5p |
| 4 | COX6B1 | hsa-miR-423-5p |  | IL1A | hsa-miR-93-5p |  | CASP3 | hsa-miR-155-5p, hsa-miR-27a-3p, hsa-miR-423-5p |
| 5 | COX6B2 | hsa-let-7a-5p, hsa-let-7c-5p |  | PIK3CA | hsa-miR-130b-3p, hsa-miR-19a-3p, hsa-miR-19b-3p, hsa-miR-93-5p |  | CASP7 | hsa-miR-15b-5p, hsa-miR-27a-3p, hsa-miR-423-5p |
| 6 | COX7B | hsa-let-7a-5p, hsa-let-7c-5p |  | PIK3CB | hsa-miR-27a-3p |  | CASP8 | hsa-miR-423-5p, hsa-miR-93-5p |
| 7 | CYC1 | hsa-miR-423-5p |  | PIK3R2 | hsa-miR-93-5p |  | CDC42 | hsa-miR-130b-3p, hsa-miR-143-3p, hsa-miR-19a-3p, hsa-miR-27a-3p, hsa-miR-423-5p, hsa-miR-93-5p |
| 8 | EIF2AK3 | hsa-miR-26a-5p |  | RXRA | hsa-miR-16-5p |  | COX7A2L | hsa-miR-143-3p, hsa-miR-155-5p, hsa-miR-93-5p |
| 9 | FAS | hsa-miR-143-3p |  | UQCRFS1 | hsa-miR-195-5p |  | CYCS | hsa-let-7c-5p, hsa-miR-423-5p, hsa-miR-93-5p |
| 10 | GSK3A | hsa-miR-423-5p |  |  |  |  | EIF2S1 | hsa-miR-423-5p, hsa-miR-93-5p |
| 11 | IKBKB | hsa-miR-423-5p |  |  |  |  | FASLG | hsa-let-7a-5p, hsa-miR-130b-3p, hsa-miR-200c-3p |
| 12 | IL1B | hsa-let-7c-5p |  |  |  |  | GSK3B | hsa-miR-143-3p, hsa-miR-195-5p, hsa-miR-93-5p |
| 13 | IL6R | hsa-let-7a-5p, hsa-let-7c-5p, hsa-miR-423-5p |  |  |  |  | LEPR | hsa-miR-423-5p, hsa-miR-93-5p |
| 14 | IRS1 | hsa-miR-143-3p |  |  |  |  | MLX | hsa-miR-200c-3p, hsa-miR-224-5p, hsa-miR-423-5p, hsa-miR-93-5p |
| 15 | IRS2 | hsa-miR-423-5p |  |  |  |  | MLXIP | hsa-let-7a-5p, hsa-let-7c-5p, hsa-miR-423-5p, hsa-miR-93-5p |
| 16 | ITCH | hsa-miR-143-3p, hsa-miR-200c-3p |  |  |  |  | NDUFA11 | hsa-let-7c-5p, hsa-miR-15b-5p, hsa-miR-93-5p |
| 17 | JUN | hsa-miR-423-5p |  |  |  |  | NDUFA5 | hsa-miR-195-5p, hsa-miR-224-5p, hsa-miR-423-5p |
| 18 | LEP | hsa-miR-200c-3p, hsa-miR-224-5p |  |  |  |  | NDUFA9 | hsa-miR-155-5p, hsa-miR-15b-5p, hsa-miR-19b-3p, hsa-miR-423-5p |
| 19 | NDUFA10 | hsa-miR-143-3p |  |  |  |  | NDUFB5 | hsa-let-7c-5p, hsa-miR-130b-3p, hsa-miR-155-5p, hsa-miR-15b-5p, hsa-miR-16-5p, hsa-miR-195-5p, hsa-miR-26a-5p, hsa-miR-423-5p |
| 20 | NDUFA12 | hsa-let-7c-5p |  |  |  |  | NDUFC2 | hsa-let-7a-5p, hsa-let-7c-5p, hsa-miR-423-5p, hsa-miR-93-5p |
| 21 | NDUFA1 | hsa-miR-143-3p |  |  |  |  | NDUFV3 | hsa-let-7a-5p, hsa-let-7c-5p, hsa-miR-19b-3p, hsa-miR-200c-3p |
| 22 | NDUFA4 | hsa-let-7a-5p, hsa-miR-15b-5p |  |  |  |  | PIK3R1 | hsa-miR-143-3p, hsa-miR-224-5p, hsa-miR-27a-3p |
| 23 | NDUFA4L2 | hsa-miR-423-5p |  |  |  |  | PRKAA2 | hsa-miR-155-5p, hsa-miR-16-5p, hsa-miR-93-5p |
| 24 | NDUFA6 | hsa-miR-423-5p |  |  |  |  | PRKAG3 | hsa-let-7c-5p, hsa-miR-143-3p, hsa-miR-15b-5p, hsa-miR-16-5p, hsa-miR-423-5p, hsa-miR-93-5p |
| 25 | NDUFB7 | hsa-miR-423-5p |  |  |  |  | SDHC | hsa-let-7c-5p, hsa-miR-15b-5p, hsa-miR-195-5p, hsa-miR-224-5p, hsa-miR-27a-3p, hsa-miR-423-5p, hsa-miR-93-5p |
| 26 | NDUFC2-KCTD14 | hsa-let-7c-5p, hsa-miR-423-5p |  |  |  |  | SREBF1 | hsa-let-7c-5p, hsa-miR-143-3p, hsa-miR-15b-5p, hsa-miR-16-5p |
| 27 | NDUFS1 | hsa-let-7a-5p, hsa-let-7c-5p, hsa-miR-15b-5p, hsa-miR-200c-3p |  |  |  |  | UQCRB | hsa-let-7a-5p, hsa-miR-15b-5p, hsa-miR-195-5p, hsa-miR-200c-3p, hsa-miR-423-5p, hsa-miR-93-5p |
| 28 | PIK3R3 | hsa-miR-21-5p, hsa-miR-423-5p |  |  |  |  |  |  |
| 29 | PKLR | hsa-miR-423-5p |  |  |  |  |  |  |
| 30 | PPARA | hsa-miR-423-5p |  |  |  |  |  |  |
| 31 | PRKAB1 | hsa-let-7a-5p, hsa-let-7c-5p, hsa-miR-26a-5p, hsa-miR-423-5p |  |  |  |  |  |  |
| 32 | PRKAB2 | hsa-miR-15b-5p |  |  |  |  |  |  |
| 33 | PRKAG1 | hsa-let-7c-5p, hsa-miR-423-5p |  |  |  |  |  |  |
| 34 | PRKAG2 | hsa-let-7c-5p, hsa-miR-15b-5p |  |  |  |  |  |  |
| 35 | RELA | hsa-miR-423-5p |  |  |  |  |  |  |
| 36 | SDHD | hsa-miR-155-5p, hsa-miR-423-5p |  |  |  |  |  |  |
| 37 | SMAD7 | hsa-miR-155-5p, hsa-miR-200c-3p, hsa-miR-423-5p |  |  |  |  |  |  |
| 38 | TGFB1 | hsa-miR-423-5p |  |  |  |  |  |  |
| 39 | TNF | hsa-miR-423-5p |  |  |  |  |  |  |
| 40 | UQCR11 | hsa-miR-143-3p |  |  |  |  |  |  |
| 41 | UQCRQ | hsa-miR-15b-5p, hsa-miR-26a-5p, hsa-miR-423-5p |  |  |  |  |  |  |
| 42 | VCAM1 | hsa-let-7c-5p |  |  |  |  |  |  |
| 43 | XBP1 | hsa-let-7a-5p, hsa-miR-423-5p |  |  |  |  |  |  |

**Supplementary Table 4.** NAFLD pathogenesis genes targeted by differentially expressed microRNAs in group of patients with NASH.
